# Multi-environment evaluations across ecological regions reveal climate and soil effects on amides contents in Chinese prickly ash peels (*Zanthoxylum bungeanum* Maxim.)

**DOI:** 10.1186/s12870-023-04328-2

**Published:** 2023-06-13

**Authors:** Tao Zheng, Hai-tao Zeng, Bing-yin Sun, Shu-ming Liu

**Affiliations:** 1 Shaanxi University of Technology, School of Biological Science and Engineering, Hanzhong, 723001 China; 2 Qinba State Key Laboratory of Biological Resources and Ecological Environment (Incubation),, Hanzhong, 723001 China; 3 Qinba Mountain Area Collaborative Innovation Center of Bioresources Comprehensive Development, Hanzhong, 723001 China; 4 Yangling Vocational &Technical College, Yangling, 712100 China; 5 College of Science, Northwest Agriculture and Forestry University, Yangling, 712100 China

**Keywords:** Chinese prickly ash, Amides, Region difference, Climatic factor, Soil factor

## Abstract

**Background:**

Environmental factors difference is the key factor for the difference in the production, transformation and accumulation of effective components in plants. UPLC-MS/MS and multivariate statistical methods were applied to describe the region difference of amides compounds in Chinese prickly ash peels from different regions and their correlation with climatic factors and soil factors.

**Results:**

Amides compounds contents were significantly higher in high altitude areas, with obvious altitude change trend. Two ecotypes were classified based on the amides compounds contents, one was the high altitude-cool type from Qinghai, Gansu, Sichuan and western Shaanxi province, and the other one was low altitude-warm type from eastern Shaanxi, Shanxi, Henan, Hebei and Shandong province. Amides compounds content were negatively correlated with annual mean temperature, max temperature of warmest month, mean temperature of wettest quarter and mean temperature of warmest quarter (*P* < 0.01). Except for hydroxy-γ-sanshool and ZP-amide A, the residual amides contents were significantly positively correlated with organic carbon, available nitrogen, phosphorus and potassium in soil and negatively correlated with soil bulk density. Low temperature, low precipitation and high organic carbon in soil were conducive to amides accumulation.

**Conclusions:**

This study aided in site specific exploration of high amides contents yielding samples, enriched the environment factors effects on amides compounds, and provided scientific foundation for the improvement of Chinese prickly ash peels quality and the location of high-quality production areas.

**Supplementary Information:**

The online version contains supplementary material available at 10.1186/s12870-023-04328-2.

## Background

Chinese prickly ash is a characteristic spice in China, mainly produced in Gansu, Sichuan, Shaanxi, Shanxi, Henan and other provinces [1, 2]. Pungent taste is the evaluation index of the main flavor substance and important quality of Chinese prickly ash peels [3, 4]. Chinese prickly ash peels could not only be used as condiment, but also has high medicinal value. Numb-taste components in Chinese prickly ash peels are chain unsaturated fatty acid amide represented by sanshool [5, 6]. It is a unique pungent and numb-taste component and is also one of the important indexes for quality identification of Chinese prickly ash peels [7, 8]. It has the effects of anesthesia, excitation, bacteriostasis, dispelling wind and removing dampness, insecticidal and analgesic [9].

The biosynthesis and accumulation of effective components in plants are affected by multiple factors of geographical environment (including precipitation, temperature, humidity, soil and other factors) [10, 11]. The formation of plant secondary metabolites is related to plants growth and development, and also is strongly regulated by environmental factors [12, 13]. Usually, altitude directly affects temperature, precipitation, sunshine hours and humidity, and its variations could influence plant growth and development and secondary metabolites accumulation [14–16]. Soil fertility and nutrient content also have important influence on the secondary metabolite contents in plants [17]. As roots are the best conduit for transferring nutrients, nutrient cycling and plant growth, soil factors play a very significant role in plant secondary metabolites production [18]. As previously mentioned, differences in soil conditions from different regions might have distinct influence on the nutrient uptake, secondary metabolites production and yield of Chinese prickly ash peels under wild conditions [19]. Therefore, to gain insight into the climate factors and soil factors effects on amides accumulation in Chinese prickly ash peels is of great significance for the effective use in amides yield optimization.

Chinese prickly ash germplasm resources are abundant in China, most of which are wild and semi-wild and concentratedly distributed in the semi-humid and semi-arid areas of the Loess Plateau and the dry and hot valley zone in the Qinba Mountains [20, 21]. Under the long-term natural selection, wild Chinese prickly ash forms different numbness characteristics [22, 23], and there was also a significant difference in amides types and contents. Moreover, similar eco-geographical conditions are prevailing and easily found in other regions in China. However, the research on the climate and soil factors across ecological regions affecting the amides accumulation in Chinese prickly ash peels were still relatively weak. The quality of Chinese prickly ash peels in those areas need to be investigated to reveal the differences of amides from a micro perspective, and the environment factors affecting the amides accumulation from a macro perspective.

In the present study, 26 wild Chinese prickly ash peels samples and 26 corresponding soil factors were collected from different regions in China. The combinations of UPLC-MS/MS and multivariate statistical approach were applied to describe the regions variation of amides in wild Chinese prickly ash peels from different regions, and to interpret the correlation between the amides compounds and ecological factors (climatic factors and soil factors). These results revealed the regional differences of amides in Chinese prickly ash peels from natural distribution area, and elucidated the key environmental factors affecting the amides accumulation, which provided a scientific basis for the protection and utilization of excellent wild Chinese prickly ash germplasm resources, the quality improvement and the selection of high-quality producing areas.

## Materials and methods

### Peels materials

Wild Chinese prickly ash fruit samples were collected fron its natural habitat in China. The mature fruits and their rhizosphere soil were collected by field investigation from 26 sites covering 8 provinces of China at different altitudes (201-2,188 m) (Each sample was collected once a year) from July to September of 2019 and 2020. The detailed sampling information was shown in Fig. S1 and Table S1. With the premise of protecting local wild Chinese prickly ash germplasm resources, representative mature fruits samples were collected from each sampling point, each containing 5 strains, which were evenly mixed together. The sampling amount was 3–5 kg, and the distance between any two sampling points was more than 50 m. All Chinese prickly ash samples were authenticated by Professor Wu Zhenhai from Northwest A & F University and deposited in College of Science, Northwest A & F University, Yangling, China. Mature peels without mechanical damage, obvious pests and diseases were dried in the shade at room temperature to constant weight (moisture content less than 10.5%), and stored at 4 °C refrigerator.

### Sample preparation and extraction

The preparation of dried Chinese prickly ash peels was based on methods published in the literature as follows [21]: (1) crushed using a mixer mill (MM 400, Retsch) for 1.5 min at 30 Hz, (2) dissolve 50 mg of powder with 1.2 mL 70% methanol solution, (3) vortex 30 s every 30 min for 6 times in total, (4) following centrifugation at 12,000 rpm for 3 min, the extracts were filtrated (SCAA-104, 0.22 μm pore size; ANPEL, Shanghai, China,) before UPLC-MS/MS analysis.

### UPLC-MS/MS analysis

The amides compounds extracts were analyzed using an UPLC-ESI-MS/MS system (UPLC, SHIMADZU Nexera X2; MS, Applied Biosystems 4500 Q TRAP) by MetWare [21]. The UPLC analytical conditions were as follows:1) column, Agilent SB-C18 (1.8 μm, 2.1 mm * 100 mm); 2) the mobile phase was consisted of solvent A, pure water with 0.1% formic acid, and solvent B, acetonitrile with 0.1% formic acid; 3) sample measurements were performed with a gradient program as follows: 0 min: 95% A, 5% B; 0–9 min: a linear gradient to 5% A, 95% B; 9–10 min: 5% A, 95% B; 10.00-11.10 min: 95% A, 5.0% B; 11.10–14.00 min: 95% A, 5.0% B; 4) flow velocity, 0.35 mL per minute, 5) column oven, 40 °C, 6) injection volume, 4 µL. ESI source operation parameters were as follows: (1) source temperature, 550 °C; (2) ion spray voltage (IS) 5500 V (positive ion mode)/-4500 V (negative ion mode); (3) ion source gas I (GSI), gas II(GSII) and curtain gas: 50, 60, and 25 psi, respectively; (4) the collision-activated dissociation (CAD), high; (5) instrument tuning and mass calibration were performed with 10 and 100 µmol/L polypropylene glycol solutions in QQQ and LIT modes, respectively; (6) QQQ scans were acquired as MRM experiments with collision gas (nitrogen) set to medium.

Analyst 1.6.3 software was applied to analyze the amides data in Chinese prickly ash peels from different regions. The peak area of amide metabolites was corrected by MultiQuant 3.02 (AB SCIEX, Concord, ON, Canada), and the corresponding relative content of amides was expressed by chromatographic peak area.

### Data on climate factors

The annual mean temperature, annual mean precipitation, annual mean sunshine hours and other meteorological data of sampling points were obtained from the Resource and Environmental Science and Data Center of the Chinese Academy of Sciences (https://www.resdc.cn/). 19 climate environment variables were obtained from the WorldClim database (version 2.1), with spatial resolution of 30s (about 1km^2^). ArcGIS 10.7 software was applied to extract climate data corresponding to the latitude and longitude data of 26 sampling sites. All the climate factors were listed in Table S2.

### Soil sample analysis

Soil bulk density and gravel contents were measured by ring knife method. Semi-micro Kjeldahl method (GB7173-87) was carried out to determine total nitrogen content, sodium hydroxide molten molybdenum antimony anti-colorimetric method (GB8937-88) was used to measure total phosphorus content, sodium hydroxide alkali fusion atomic absorption spectrometry was used for determination of total potassium content (GB9836-88), and soil organic carbon content was determined by combustion oxidation-titration (HJ 658–2013) [17]. The amount of ammonium ion was determined by distillation method and converted into soil cation exchange capacity. Soil pH and moisture tester was applied to determine of soil pH. The soil factors were listed in Table S3.

### Data analysis

Chemometric analyses such as hierarchical cluster analysis (HCA), principal component analysis (PCA), orthogonal partial least squares-discriminant analysis (OPLS-DA), correlation analysis (CA), path analysis (PA) and structural equation model (SEM), were employed to systematically analyze the geographical variation of amides contents in Chinese prickly ash peels from different regions and the relationship between environmental factors and amides contents. Among them, HCA, PCA, OPLS-DA and CA were generated using the Origin software for statistical and computing (Origin Pro 2020b, Origin Lab, USA), and PA and SEM were performed using SPSS 24.0 for Windows (SPSS Inc., Chicago, IL, USA).

## Results

### Determination of amides contents in Chinese prickly ash peels

**Fig. 1:**
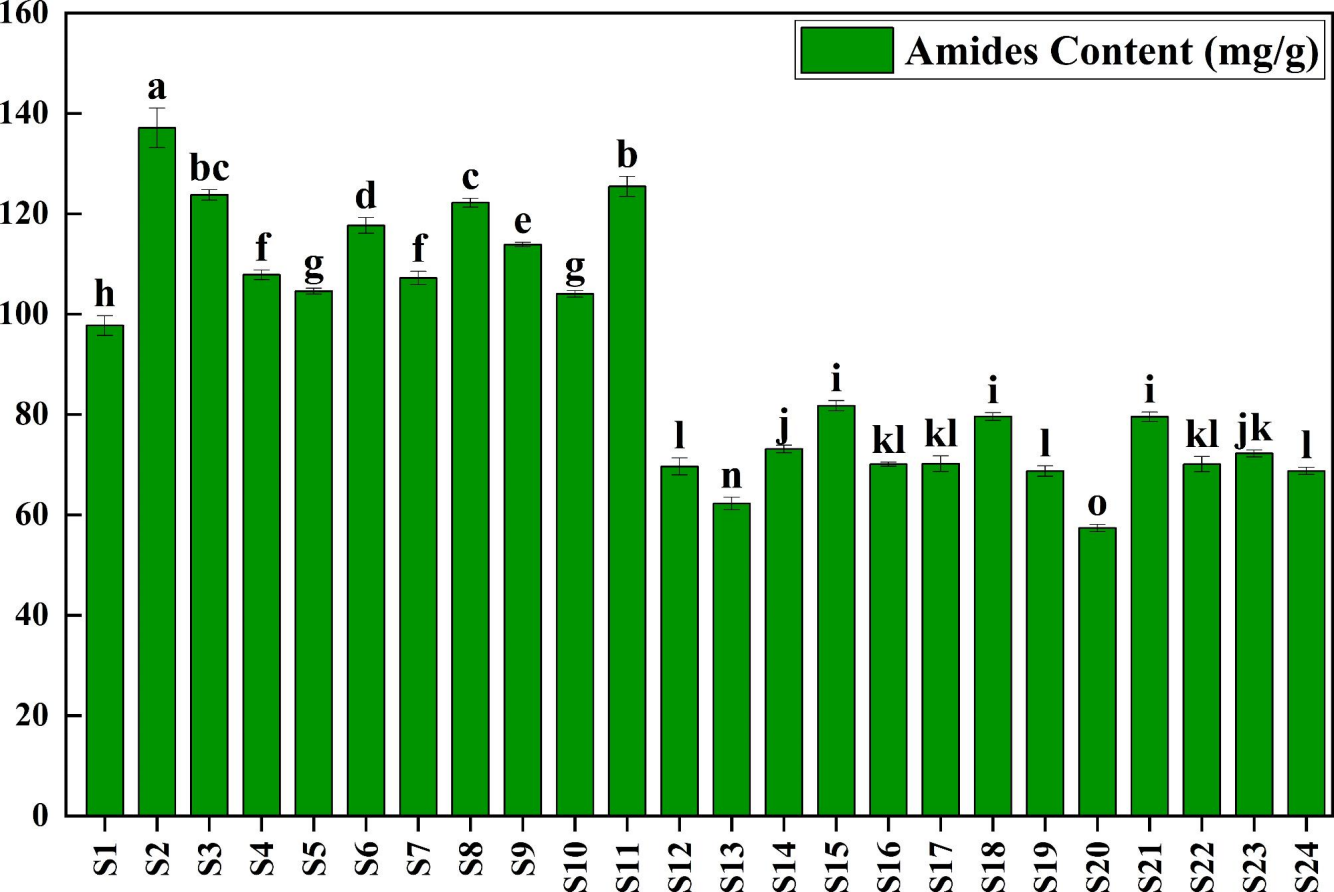
Amides contents in Chinese prickly ash peels from different regions. Different lowercase letters represented significant difference at 0.05 level (*P* < 0.05)

Significant differences in the total amide contents in Chinese prickly ash peels were obviously observed (Fig. 1). The amides contents were 57.38 to 137.11 mg/g, with the average content of 88.92 mg/g. The amides contents in peels from S2 (Hanyuan County), S5 (Linxia County), S8 (Wudu District), S9 (Qin’an County) and S11 (Fengxian County) were the higher. The variation coefficient of amide content was 26.89%, which revealed that there were abundant regional differences in total amide contents. The total amides contents in peels from high altitude areas were obviously higher than that in low altitude area, which indicated that the total amide contents were negatively correlated with altitude.

### Qualitative and quantitative analysis of amides in Chinese prickly ash peels

UPLC-MS/MS was applied to determine amide compounds in Chinese prickly ash peels from different rehions, and 13 amide compounds were identified, including γ-sanshool, ZP-amide N, dihydrosanshool, tetrahydrosanshool, bungeanool, hydroxy-β-sanshool, hydroxy-α-sanshool, hydroxy-γ-sanshool, dehydroxy-γ-sanshool, ZP-amide C, ZP-amide J, ZP-amide M, ZP-amide A. Among them, ZP-amide C was not detected in S12, S14, S15, S16, S17, S21 and S22, and dehydroxy-γ-sanshool and ZP-amide J were not detected in S18 and S20 (Table 1).

The main amides in Chinese prickly ash peels were hydroxy-α-sanshool (24.38–66.24 mg/g), hydroxy-β-sanshool (8.34–12.39 mg/g) and hydroxy-γ-sanshool (3.27–15.45 mg/g), with the average contents of hydroxy-α-sanshool, hydroxy-β-sanshool and hydroxy-γ-sanshool were 44.14 mg/g, 10.77 mg/g and 7.18 mg/g, respectively. The contents of hydroxy-α-sanshool, hydroxy-β-sanshool and hydroxy-γ-sanshool accounted for 74.06% of the total amount of amides. The hydroxy-α-sanshool content was the highest, which was the major amide compound to determine the numb taste of Chinese prickly ash peels. Among them, the hydroxy-α-sanshool contents of S8, S9, S11, S2 and S3 were higher than 63.00 mg/g, and hydroxy-α-sanshool contents of S20 and S24 were the lowest, with the content of 24.97 ± 0.31 mg/g and 24.38 ± 0.49 mg/g, respectively. The hydroxy-β-sanshool contents of S1, S6 and S11 were the highest, which were 12.39 ± 0.31,12.62 ± 0.35 and 11.82 ± 0.33 mg/g, respectively, whereas that of S7, S18 and S24 were the lowest. The hydroxy-γ-sanshool contents in S22, S23 and S24 were the highest, which were 15.45 ± 0.36,12.41 ± 0.16 and 12.09 ± 0.25 mg/g, respectively. The hydroxy-γ-sanshool contents in S1, S2 and S3 were 3.27 ± 0.22,4.20 ± 0.16 and 3.44 ± 0.09 mg / g, respectively. The γ-sanshool content was 1.25 to 7.68 mg/g, with the average content of 2.99 mg/g. ZP-amide N content was 0.05 to 6.25 mg/g, with an average content of 1.87 mg/g. The content of dihydrosanshool was 0.34 to 4.43 mg/g, with an average content of 2.67 mg/g. Tetrahydrosanshool content was 0.48 to15.35 mg/g, and the average content was 6.30 mg/g. Bungeanool content was 0.38 to 5.23 mg/g, and the average content was 2.84 mg/g. Dehydroxy-γ-sanshool content was 0.84 to 7.50 mg/g, and the average content was 2.52 mg/g. ZP-amide C content was 2.72–8.96 mg/g, and the average content was 4.34 mg/g. ZP-amide J ranged from 1.12 to 2.56 mg / g, and the average content was 1.76 mg/g. The average content of ZP-amide M was 0.97 mg/g, ranging from 0.05 to 2.71 mg /g. ZP-amide A ranged from 0.11 to 1.56 mg/g, with an average content of 0.56 mg/g.

The variation coefficient of 13 amides ranged from 12.07 to 86.88%, with the average variation coefficient of 53.91%. The variation coefficients of ZP-amide N, tetrahydrosanshool, ZP-amide C, ZP-amide M and ZP-amide A were higher than 64%, which indicated that the differences of amide compounds in Chinese prickly ash peels from different regions were larger. The variation range of hydroxy-β-sanshool was 12.07%, which demonstrated that the hydroxy-β-sanshool content in Chinese prickly ash peels from different regions was stable.

**Table 1:** Amides compounds contents in Chinese prickly ash peels from different regions

Code	Amides compounds content (mg/g)												
	Y_γSS_	Y_AN_	Y_DDBG_	Y_TDBG_	Y_BG_	YHβSS	Y_HαSS_	Y_HγSS_	Y_DHγSS_	Y_AC_	Y_AJ_	Y_AM_	Y_AA_
S1	4.47 ± 0.08^e^	3.35 ± 0.12^e^	3.42 ± 0.11^e^	9.93 ± 0.27^e^	4.75 ± 0.32^b^	12.39 ± 0.31^ab^	42.96 ± 1.62^f^	3.27 ± 0.22^n^	2.27 ± 0.22^ fg^	5.38 ± 0.15^ g^	1.86 ± 0.11^f^	2.71 ± 0.14^a^	0.95 ± 0.10^d^
S2	7.68 ± 0.23^a^	6.25 ± 0.23^a^	2.98 ± 0.05^ g^	16.35 ± 0.42^a^	5.23 ± 0.09^a^	10.67 ± 0.55^de^	66.72 ± 2.13^a^	4.20 ± 0.16^ m^	3.66 ± 0.15^d^	7.81 ± 0.23^b^	1.71 ± 0.12^ g^	2.63 ± 0.13^a^	1.20 ± 0.05^b^
S3	6.14 ± 0.21^b^	3.49 ± 0.07^d^	3.16 ± 0.15^ fg^	13.02 ± 0.43^b^	5.06 ± 0.14^a^	10.94 ± 0.36^d^	61.95 ± 0.96^c^	3.44 ± 0.09^n^	3.56 ± 0.07^d^	6.80 ± 0.16^d^	2.55 ± 0.27^a^	2.07 ± 0.21^b^	1.56 ± 0.05^a^
S4	4.56 ± 0.13^de^	3.08 ± 0.06^f^	3.74 ± 0.11^d^	9.51 ± 0.08^f^	3.77 ± 0.12^d^	11.75 ± 0.59^ab^	54.97 ± 1.48^e^	6.24 ± 0.03^i^	2.31 ± 0.15^f^	4.33 ± 0.04^i^	1.62 ± 0.12^ h^	0.94 ± 0.01^f^	0.99 ± 0.06^d^
S5	4.64 ± 0.07^de^	3.05 ± 0.06^f^	3.65 ± 0.03^d^	9.10 ± 0.12^ g^	3.72 ± 0.13^de^	11.53 ± 0.81^c^	53.91 ± 0.19^e^	5.06 ± 0.13^k^	2.02 ± 0.11^ h^	4.50 ± 0.08^i^	1.67 ± 0.05^ h^	0.99 ± 0.07^e^	0.74 ± 0.02^f^
S6	4.75 ± 0.09^d^	2.78 ± 0.06^ g^	4.53 ± 0.09^a^	12.08 ± 0.31^c^	4.16 ± 0.19^c^	12.62 ± 0.35^a^	58.49 ± 1.28^d^	4.13 ± 0.09^ m^	2.01 ± 0.14^ h^	7.27 ± 0.13^c^	1.72 ± 0.010^ fg^	2.05 ± 0.06^b^	1.13 ± 0.09^c^
S7	4.53 ± 0.07^de^	3.02 ± 0.04^f^	3.19 ± 0.09^f^	11.46 ± 0.05^d^	3.87 ± 0.09^d^	8.58 ± 0.26^ g^	60.75 ± 1.25^c^	4.18 ± 0.11^ m^	2.25 ± 0.09^ fg^	3.77 ± 0.15^j^	1.47 ± 0.05^i^	0.024 ± 0.04^k^	0.11 ± 0.04^ L^
S8	5.36 ± 0.41^c^	3.98 ± 0.04^c^	4.40 ± 0.05^a^	10.87 ± 0.56^e^	4.82 ± 0.07^b^	10.81 ± 0.41^d^	64.98 ± 1.24^ab^	5.48 ± 0.12^jk^	3.28 ± 0.05^e^	5.04 ± 0.09^ h^	2.56 ± 0.05^a^	0.47 ± 0.02 h	0.14 ± 0.03^ L^
S9	3.60 ± 0.08f	4.06 ± 0.07^c^	3.99 ± 0.06^c^	8.87 ± 0.07^ g^	3.56 ± 0.19^e^	11.74 ± 0.29^ab^	64.79 ± 0.27^b^	4.71 ± 0.07^ L^	3.87 ± 0.11^c^	2.72 ± 0.11^ L^	1.24 ± 0.16^j^	0.52 ± 0.02^ g^	0.17 ± 0.02^kl^
S10	3.23 ± 0.13 h	1.91 ± 0.08^ h^	3.04 ± 0.05^ fg^	7.47 ± 0.28^ h^	4.11 ± 0.06^c^	11.15 ± 0.44^ cd^	60.65 ± 0.98^c^	4.05 ± 0.10^ m^	3.34 ± 0.13^e^	2.98 ± 0.13^k^	1.12 ± 0.05^k^	0.53 ± 0.04 g	0.48 ± 0.01^ h^
S11	3.44 ± 0.02^ g^	4.25 ± 0.13^b^	3.19 ± 0.04^ fg^	10.88 ± 0.24^e^	5.08 ± 0.06^a^	11.82 ± 0.33^ab^	66.24 ± 1.51^ab^	5.74 ± 0.18j	3.67 ± 0.31^d^	7.89 ± 0.18^b^	1.32 ± 0.03^ij^	1.43 ± 0.05^c^	0.45 ± 0.02^hi^
S12	2.16 ± 0.06^j^	0.78 ± 0.02^j^	1.78 ± 0.05^k^	3.56 ± 0.09^k^	1.82 ± 0.04^i^	9.71 ± 0.28^ef^	38.26 ± 1.44^gh^	5.62 ± 0.09^jk^	2.06 ± 0.04^gh^	—	2.38 ± 0.03^b^	1.16 ± 0.05^d^	0.34 ± 0.01^j^
S13	1.61 ± 0.02^ m^	0.79 ± 0.01^j^	1.53 ± 0.05^ L^	1.66 ± 0.04^ L^	0.94 ± 0.04^k^	9.76 ± 0.33^ef^	27.62 ± 1.05^k^	5.30 ± 0.04^jk^	1.44 ± 0.08^j^	7.71 ± 0.28^b^	2.24 ± 0.04^bc^	1.30 ± 0.06^ cd^	0.352 ± 0.03^j^
S14	1.43 ± 0.10^n^	0.42 ± 0.02^ L^	1.69 ± 0.05^k^	1.98 ± 0.08^ L^	1.46 ± 0.02^j^	11.66 ± 0.37^b^	39.94 ± 0.28^ g^	7.28 ± 0.19^gh^	4.32 ± 0.10^b^	—	1.99 ± 0.03^de^	0.48 ± 0.02^ h^	0.38 ± 0.06^ij^
S15	1.52 ± 0.02^mn^	0.52 ± 0.01^ L^	1.92 ± 0.09^j^	1.76 ± 0.05^ L^	1.42 ± 0.10j	12.83 ± 0.28^a^	43.49 ± 0.78^f^	7.62 ± 0.27^ g^	7.50 ± 0.06^a^	—	2.22 ± 0.11^c^	0.58 ± 0.018 g	0.35 ± 0.01^j^
S16	1.45 ± 0.03^mn^	0.53 ± 0.03^ L^	2.32 ± 0.09^i^	3.42 ± 0.08^k^	2.52 ± 0.25	11.39 ± 0.30^c^	36.32 ± 0.51^i^	8.15 ± 0.01^f^	0.84 ± 0.03^k^	—	1.83 ± 0.06^f^	0.99 ± 0.06^e^	0.34 ± 0.01^j^
S17	1.89 ± 0.04^kl^	0.61 ± 0.01^kl^	2.17 ± 0.08^ij^	4.31 ± 0.12^ij^	2.11 ± 0.14^ h^	11.46 ± 0.19^c^	35.24 ± 1.04^i^	7.34 ± 0.21^gh^	1.65 ± 0.02^i^	—	1.77 ± 0.07^ fg^	1.26 ± 0.14^ cd^	0.40 ± 0.06^i^
S18	1.49 ± 0.01^mn^	0.60 ± 0.02^kl^	1.99 ± 0.13^ij^	1.64 ± 0.02^ L^	2.80 ± 0.09^f^	11.07 ± 0.10^ cd^	41.71 ± 0.53^f^	10.98 ± 0.07^c^	—	6.03 ± 0.11^f^	—	1.04 ± 0.01^e^	0.24 ± 0.04^k^
S19	1.78 ± 0.02^ L^	0.60 ± 0.01^kl^	2.10 ± 0.03^ij^	4.05 ± 0.07^ij^	1.77 ± 0.06^i^	8.34 ± 0.12^ g^	33.28 ± 0.75^j^	7.06 ± 0.12^ h^	2.23 ± 0.08^ fg^	5.28 ± 0.08^ g^	1.66 ± 0.04^ h^	0.23 ± 0.01^i^	0.32 ± 0.01^j^
S20	2.01 ± 0.08^k^	0.80 ± 0.05^j^	2.35 ± 0.08^hi^	4.15 ± 0.08^ij^	1.84 ± 0.01^hi^	9.20 ± 0.20^ fg^	24.97 ± 0.31^ L^	6.09 ± 0.06^i^	—	5.34 ± 0.10^ g^	—	0.27 ± 0.01^i^	0.36 ± 0.01^j^
S21	2.28 ± 0.05^i^	0.99 ± 0.02^i^	4.27 ± 0.06^b^	4.14 ± 0.30^ij^	2.47 ± 0.04^ g^	10.21 ± 0.08^e^	37.52 ± 0.61^ h^	10.54 ± 0.22^d^	3.37 ± 0.08^e^	—	2.31 ± 0.05^bc^	1.08 ± 0.05^de^	0.36 ± 0.02^j^
S22	2.16 ± 0.02^j^	0.99 ± 0.08^i^	2.55 ± 0.03^ h^	4.49 ± 0.01^i^	2.04 ± 0.06^hi^	9.45 ± 0.25^f^	27.49 ± 1.27^k^	15.45 ± 0.36^a^	1.44 ± 0.01^j^	—	2.05 ± 0.12^d^	1.21 ± 0.05^d^	0.78 ± 0.03^ef^
S23	1.38 ± 0.02^o^	0.65 ± 0.02^k^	2.23 ± 0.07^i^	4.13 ± 0.07^ij^	1.90 ± 0.04^hi^	9.45 ± 0.15^f^	25.67 ± 0.58^ L^	12.41 ± 0.16^b^	2.32 ± 0.03^f^	8.96 ± 0.07^a^	1.77 ± 0.04^ fg^	0.92 ± 0.16^f^	0.45 ± 0.05^hi^
S24	1.25 ± 0.04^o^	0.51 ± 0.02^ L^	2.42 ± 0.12^hi^	3.96 ± 0.05^j^	1.85 ± 0.04^hi^	8.52 ± 0.23^ g^	24.38 ± 0.49^ L^	12.09 ± 0.25^b^	2.09 ± 0.11^gh^	8.12 ± 0.07^b^	2.45 ± 0.05^ab^	0.29 ± 0.01^i^	0.82 ± 0.02^e^
S25	1.60 ± 0.08^ m^	0.51 ± 0.05^ L^	0.56 ± 0.02^ m^	0.59 ± 0.01^ m^	0.38 ± 0.02^ L^	11.35 ± 1.16^c^	25.97 ± 0.51^kl^	9.95 ± 0.42^e^	2.19 ± 0.16^ g^	6.63 ± 0.19^d^	2.41 ± 0.07^ab^	0.05 ± 0.02^j^	0.46 ± 0.02^hi^
S26	1.25 ± 0.07^o^	0.05 ± 0.00^ m^	0.34 ± 0.01^n^	0.48 ± 0.03^ m^	0.46 ± 0.02^ L^	11.55 ± 0.22^c^	29.45 ± 1.36^j^	10.39 ± 0.33^d^	1.78 ± 0.11^i^	6.35 ± 0.07^e^	1.96 ± 0.01^e^	0.07 ± 0.01^j^	0.59 ± 0.02^ g^
Mean	2.99	1.87	2.67	6.30	2.84	10.77	44.14	7.18	2.52	4.34	1.76	0.97	0.56
CV/%	58.50%	86.88%	39.86%	68.53%	51.52%	12.07%	33.60%	43.62%	58.39%	69.77%	36.57%	77.38%	64.10%

Note: ‘—’ was not detected. Y_γSS_-γ-Sanshool, Y_AN_-ZP-amide N, Y_DHγSS_-Dehydro-γ-sanshool, Y_DDBG_-Dihydrobungeanool, Y_TDBG_-Tetrahydrobungeanool, Y_BG_-Bungeanool, Y_HβSS_-Hydroxy-β-sanshool, Y_HαSS_-Hydroxy-α-sanshool, Y_HγSS_-Hydroxy-γ-sanshool, Y_AC_-ZP-amide C, Y_AJ_-ZP-amide J, Y_AM_-ZP-amide M, Y_AA_-ZP-amide A. Each different letters in the same column in the table indicated that amines contents in Chinese prickly ash peels were significantly different (*P* < 0.05), and the same letter indicated that there was no significant difference (*P* > 0.05)

### Difference analysis of amides in Chinese prickly ash peel from different regions

To further elucidate the regional difference of amide compounds contents in Chinese prickly ash peels from different regions, chemometric analyses such as hierarchical cluster analysis (HCA), principal component analysis (PCA), and orthogonal partial least squares-discriminant analysis (OPLS-DA) were applied to illustrate the region variation of amides and screen out the key amide substances that distinguish and evaluate Chinese prickly ash peels from different regions.

#### PCA analysis

PCA and HCA were performed to clarify the differences and similarities of amide compounds in Chinese prickly ash peels from different region, and 13 kinds of amides were used as variables. The eigenvalues of the first three principal components were greater than 1, and the cumulative variance contribution rate reached 74.720% (PC1-50.158%, PC2-13.558%, PC3-11.004%), indicating that 13 amides could distinguish Chinese prickly ash peels from different regions. The PCA results were shown in Fig. S2A. In the 2D plot, the distribution of 26 samples was more dispersed on the score diagram of the principal component, indicating that the amides contents in peels from different regions were quite different, and 26 samples were obviously clustered into 4 groups. Among them, S7, S8, S9, S10 and S11 were grouped together. Both of them came from Qinling-Bashan Mountain area. Samples S1, S2, S3, S4, S5 and S6 were clustered into a group, indicating that the amides in peels from high altitude areas (Guide, Xunhua, Hanyuan, Maoxian, Linxia) was similar. Samples S12, S14, S15, S16, S17, S18 and S19 were gathered into a larger group. The remaining samples have the largest clusters on the negative axis of PC2, most of which were from Shandong Province, indicating that the amides content in peels from Shandong Province were quite different from that in other regions. Since the variance contribution rate of PC1 was markedly higher than that of PC2, it could be inferred that amide compounds contents in Chinese prickly ash peels located in the first quadrant were higher. Therefore, the peels of Wudu, Wenxian, Qin’an and Fengxian had strong numbness and excellent in quality.

#### HCA analysis

The furthest neighoor’s method was put in application to sort samples into groups, and the pearson correlation distance was selected as the similarity measure standard, and the HCA results were described in Fig. S2B. The HCA results revealed that 26 samples were clustered into 4 groups, which was consistent with the PCA results. The first group included S1, S2, S3, S4, S5 and S6. Both of them were located in high altitude areas and the γ-sanshool, ZP-amide N, dihydrosanshool, bungeanool and ZP-amide C contents were higher. The second group was composed of samples S7, S8, S9, S10 and S11, which came from Qinling-Bashan Mountain areas. The contents of hydroxy-β-sanshool and hydroxy-α-sanshool were higher than that of all other samples. Samples S12, S14, S15, S16, S17, S18 and S19 were divided into the third group. The contents of total amides, γ-sanshool, bungeanool, hydroxy-β-sanshool and hydroxy-α-sanshool were similar, and they were all located at the junction of Henan, Shaanxi and Shanxi provinces. The last group was the remaining samples, which largely came from Hebei and Shandong provinces. The hydroxy-β-sanshool and hydroxy-α-sanshool contents were lower, and the hydroxy-γ-sanshool content was highest. HCA results demonstrated that the samples clustered into one group indicated that the synthesis and accumulation pathways of the amides in peels were similar, and the pungent taste was similar. The samples were not clustered completely based on the classification of variety groups, which indicated that they were affected by the growing locations, environmental factors. Moreover, each group had a geographical continuity.

#### OPLS-DA analysis

Although the PCA method could effectively extract the main information, it is not sensitive to variables with low correlation. OPLS-DA analysis is a multivariate statistical analysis method for supervised pattern recognition, which combined orthogonal signal correction and partial least squares-discriminant analysis to maximize the distinction between groups and could screen out the key differential compounds through variable importance projection (VIP).

**Fig. 2:**
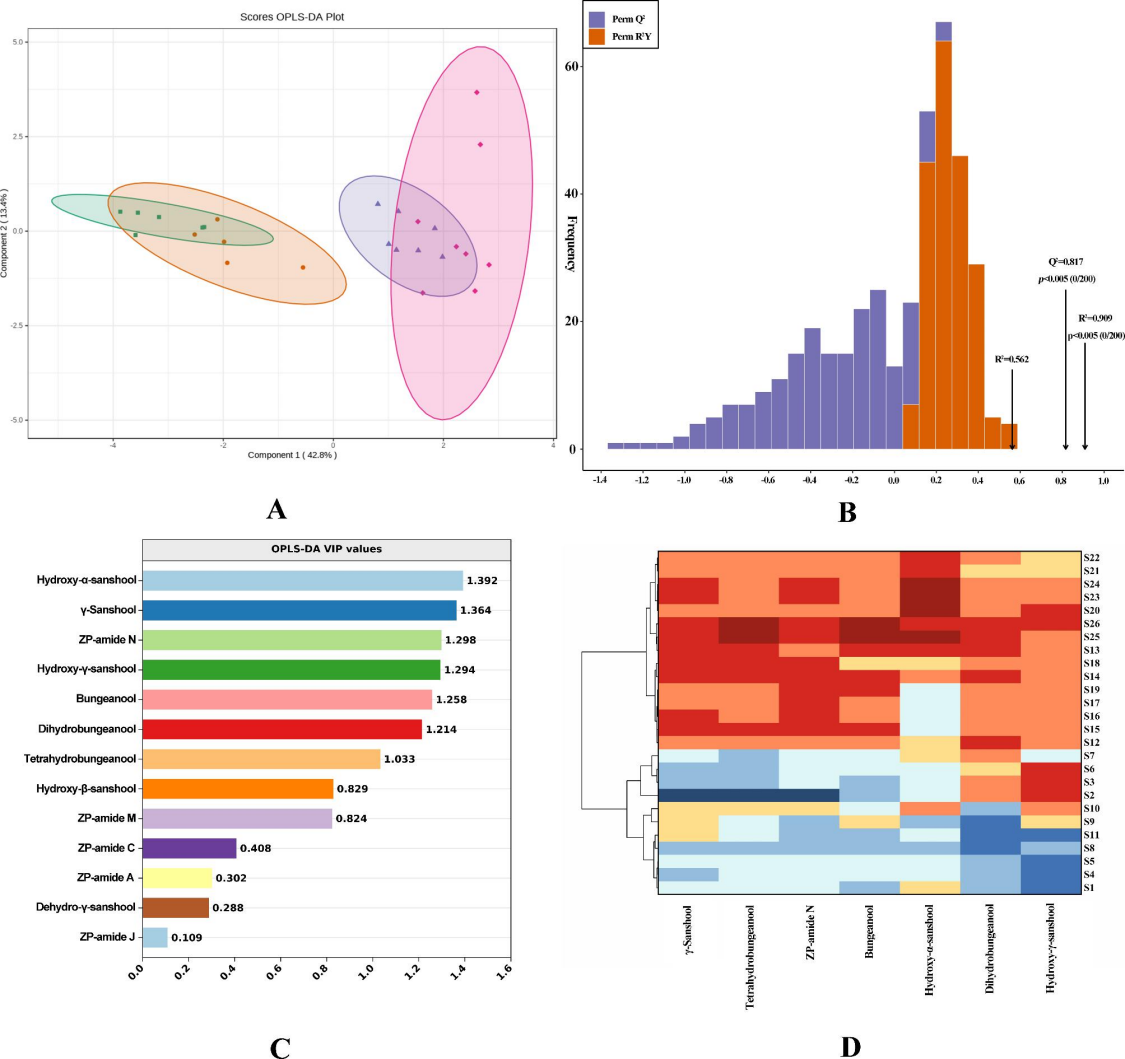
OPLS-DA analysis of Chinese prickly ash peels based on amides compounds. **A** OPLS-DA scatter plot. **B** 200 permutation tests. **C** VIP value of differential compounds. **D** Cluster heat map based on key differential compounds

The OPLS-DA score scatter diagram of Chinese prickly ash peels from different regions displayed that 26 samples was distinguished at the first principal component (42.8%), and could be divided into two groups (Fig. 2A). The first group was mainly from high altitude areas (Gansu, Qinghai, Sichuan, western Shaanxi), and the second group was mainly from low altitude areas (eastern Shaanxi, Henan, Hebei, Shanxi, Shandong Provinces). These results indicated that there were significant differences in amide compounds contents in peels, and 26 samples from different regions could be grouped into high altitude type and low altitude type.

Permutation test was mainly used to verify the fitting degree of OPLS-DA model. The explanatory ability and predictive ability of the established model were represented by R^2^Y and Q^2^ respectively. The closer the values of R^2^Y and Q^2^ were to 1, the stronger the explanatory ability and predictive ability of the model were. The model performed 200 random permutation and combination experiments on the data. R^2^Y and Q^2^ reached 90.9% and 81.7%, respectively, indicating that the OPLS-DA model has strong discrimination and prediction ability (Fig. 2B). In addition, in the permutation test, the points on the left side of R^2^ Y and Q^2^ were lower than those on the right side, and all the points of R^2^ Y were higher than those of Q^2^, indicating that the model was stable and reliable, and there was no over-fitting phenomenon. The established model could be used for subsequent screening of key differential compounds.

Based on the results of OPLS-DA, the key amide compounds were effectively screened out to distinguish Chinese prickly ash peels from different habitats. According to the VIP value (VIP > 1) of the OPLS-DA model, 7 amide compounds with greater contribution to the differentiation were selected (Fig. 2C). It can be seen that hydroxy-α-sanshool (VIP = 1.392) had the largest contribution rate to the discrimination, followed by γ-sanshool (VIP = 1.364), ZP-amide N (VIP = 1.298), hydroxy-γ-sanshool (VIP = 1.294), bungeanool (VIP = 1.258), tetrahydrosanshool (VIP = 1.214) and dihydrosanshool (VIP = 1.033). VIP score showed that these 7 substances were characteristic amide substances that distinguished Chinese prickly ash peels from different regions. The cluster heatmap plot of 26 samples was drawn with 7 key amides compounds screened, and the heatmap exhibited that 26 samples were obviously divided into two groups (Fig. 2D). Hydroxy-α-sanshool, γ-sanshool, ZP-amide N, bungeanool, tetrahydrosanshool and dihydrosanshool had higher content in peels from high altitude areas. The seven key amides contents might play an important role in the formation of unique flavor of Chinese prickly ash peels from different habitats.

### Environmental factors driving geographical variation of amides compounds

#### Correlation analysis between climatic factors and amides

**Fig. 3:**
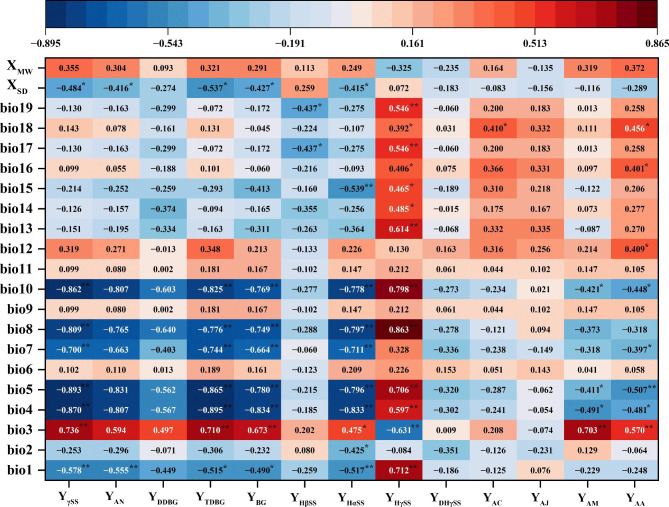
Correlation analysis results of climate factors and amides compounds Note: Y_γSS_, Y_AN_, Y_DHγSS_, …, Y_AA_ were performed in Table 1. X_MW_ (m/s)-Mean wind speed, X_SD_ (h)-Annual sunshine duration, bio1- Annual Mean Temperature (℃), bio2- Mean Diurnal Range (℃), bio3- Isothermality, bio4-Temperature Seasonality (℃), bio5-Max Temperature of Warmest Month (℃), bio6-Min Temperature of Coldest Month (℃), bio7-Temperature Annual Range (℃), bio8-Mean Temperature of Wettest Quarter (℃), bio9-Mean Temperature of Driest Quarter (℃), bio10-Mean Temperature of Warmest Quarter (℃), bio11-Mean Temperature of Coldest Quarter (℃), bio12-Annual Precipitation (mm), bio13-Precipitation of Wettest Month (mm), bio14-Precipitation of Driest Month (mm), bio15-Precipitation Seasonality (mm), bio16-Precipitation of Wettest Quarter (mm), bio17-Precipitation of Driest Quarter (mm), bio18-Precipitation of Warmest Quarter (mm), bio19-Precipitation of Coldest Quarter (mm), X_SD_-Annual sunshine duration(h), X_MW_-Mean wind speed (m/s).** represented significant difference at 0.01 level, and * represented significant difference at 0.05 level.

The production and accumulation of amides contents in Chinese prickly ash peels from different regions were regulated by climate factors. The correlation analysis results exhibited that there were different degrees of correlation between amides and climatic factors, and the results were shown in Fig. 3. Amide compounds were negatively correlated with bio1(annual mean temperature). Y_γSS_, Y_AN_, Y_DDBG_, Y_TDBG_, Y_BG_ and Y_HαSS_ were negatively correlated with bio1, bio4 (temperature seasonality), bio5 (max temperature of warmest month), bio7 (temperature annual range), bio8 (mean temperature of wettest quarter) and bio10 (mean temperature of warmest quarter) (*P* < 0.01), and positively correlated with bio3 (isothermality) (*P* < 0.01), which suggested that high temperature and temperature fluctuation were not conducive to amides formation. Y_γSS_, Y_AN_, Y_DDBG_, Y_BG_ and Y_HαSS_ exhibit a negative correlation with annual sunshine duration (*P* < 0.05). Except for Y_HβSS_, the remaining amides compounds were positively correlated with bio12 (annual precipitation). Y_HγSS_ was positively correlated with bio1, bio4, bio5, bio8 and bio10, and negatively correlated with bio3, indicating that high temperature in the growing season was conducive to the Y_HγSS_ biosynthesis. Y_AM_ and Y_AA_ displayed a positive correlation with bio3 (*P* < 0.05), and a negative correlation with bio4, bio5 and bio10 (*P* < 0.05). Y_AA_ was positively correlated with bio12, bio16 and bio18 (*P* < 0.05), indicating that low temperature and sufficient precipitation were conducive to the Y_AA_ accumulation. Most of the amides were positively linked to wind speed. In short, the low temperature and less precipitation from coloring period to mature period were conducive to amides compounds accumulation, and could help to improve the Chinese prickly ash peels quality.

In addition, the correlation between climatic factors and amides contents suggested that the climatic conditions in Hanyuan, Wudu, Qin’an, Wenxian and Fengxian were conducive to the amides accumulatio. In order to produce Chinese prickly ash peels with excellent quality, the semi-humid and semi-arid areas in the Qinba Mountains and the hilly and gully areas of the Loess Plateau (with an annual mean temperature of 11 to15°C, an annual mean precipitation of 400 to 600 mm, and a growth period of sunshine duration not less than 1,200 h) should be selected as high-quality producing areas for large scale cultivation of Chinese prickly ash.

#### Correlation analysis between soil factors and amides

Different soil physicochemical properties also had significant effect on the accumulation of amides compounds in Chinese prickly ash peels. Y_γSS_, Y_AN_, Y_DDBG_, Y_TDBG_, Y_BG_ and Y_HαSS_ were positively correlated with X_CEC_, X_N_, X_K_, X_P_ and X_SOC_ (*P* < 0.01), and negatively correlated with X_BD_ (*P* < 0.01) (Fig. S3). Y_AM_ and Y_AA_ were positively correlated with X_CEC_ (*P* < 0.01), and Y_AA_ was negatively correlated with X_PH_, X_BD_ and X_TH_ (*P* < 0.05). In summary, it demonstrated that soil total N, P, K, soil organic carbon, cation exchange capacity and soil bulk density had a great effect on amides accumulation. The soil PH value of sampling sites had no significant effect on amides accumulation.

#### Path analysis

To gain insight into the response characteristics between environmental factors and amide compounds content, path analysis (PA) was performed to calculate the direct and indirect effects of environmental factors on amide content. Taking 13 kinds of amides as independent variables and environmental factors as dependent variables, the direct and indirect path coefficients of dominant environmental factors were calculated by stepwise regression analysis.

The PA results of (Table 2) in this study demonstrated the environmental factors had significant effects on amide compounds content. It could be seen that the indirect effects of environmental factors on amide compounds were generally weaker than its direct effect, except for bio10 on Y_TDBG_, X_P_ and bio 7 on Y_HαSS_, and X_N_ on Y_HγSS_, indicating that the environmental factors had direct and decisive effects on the biosynthesis and accumulation of amide compounds.

X_N_ had a positive direct effect (0.659) on Y_HαSS_, and a negative direct effect (-0.54) Y_HγSS_. X_MW_ had a positive direct effect (0.173) on Y_γSS_, but with the negative indirect effect (-0.182) and the positive correlation coefficient (0.355), suggesting that the indirect effect X_MW_ on Y_γSS_ through bio5 and bio4 was the contributory cause of relevance. Bio4 had both negative direct and indirect effects on Y_γSS_ and Y_TDBG_, but with a positive indirect effect (0.232) on Y_BG_. Bio5 had both negative direct and indirect effects on Y_γSS_ and Y_AN_ (*P* < 0.05), and the direct effects were higher than the indirect effect, which demonstrated that bio5 played a direct and decisive role in Y_γSS_ and Y_AN_ accumulation. Bio17 had negative direct effects on Y_HβSS_ and Y_HαSS_ (*P* < 0.05), but to Y_HαSS_, displayed a positive indirect effect. X_SOC_ was the key environment factor for the contents of YAN and Y_HαSS_ (*P* < 0.05). X_K_ was the key factor for Y_DHγSS_, with a positive direct effect (0.451), and X_CEC_ was the key factor for Y_AA_, and had a positive direct effect (0.651). In a word, path analysis illustrated the relative contribution rate of various environmental factors to amides, which made the multivariate statistical analysis more rational.

**Table 2:** Path analysis between environmental factors and amides compounds content in Chinese prickly ash peels

Item		Factors	Correlation	Direct Path	Indirect Path coefficient					Decision	Significance level
			Coefficients	Coefficients						coefficient	P-value
YγSS					Total	→bio5	→bio4	→X_MW_			
		bio5	-0.893	-0.527	-0.366		-0.331	-0.035		0.663	0.001
		bio4	-0.870	-0.396	-0.474	-0.441		-0.033		0.532	0.009
		X_MW_	0.355	0.173	-0.182	-0.106	-0.076			0.093	0.034
Y_AN_					Total	→bio5	→X_SOC_				
		X_bio5_	-0.831	-0.469	-0.362		-0.363			0.560	0.025
		X_SOC_	0.825	0.431	0.394	0.395				0.525	0.038
Y_DDBG_					Total	→X_N_	→bio2	→bio6	→bio9		
		X_N_	0.649	1.343	-0.694		-1.455	1.021	-0.260	-0.060	0.000
		bio2	-0.071	2.695	-2.766	-0.725		-4.380	2.339	-7.646	0.000
		bio6	0.013	5.461	-5.448	0.253	-2.161		-3.540	-29.681	0.003
		bio9	0.002	-3.657	3.659	0.095	-1.722	5.286		-13.388	0.007
Y_TDBG_					Total	→bio4	→bio10				
		bio4	-0.895	-0.626	-0.269		-0.269			0.729	0.000
		bio10	-0.825	-0.365	0.460	0.46				0.469	0.004
Y_BG_					Total	→bio4	→bio12				
		bio4	-0.834	-1.066	0.232		0.232			0.642	0.000
		bio12	0.213	-0.402	0.615	0.615				-0.333	0.002
Y_HβSS_											
		bio17	-0.437	-0.437						0.191	0.026
Y_HαSS_					Total	→X_SOC_	→X_P_	→bio17	→bio7		
		X_SOC_	0.910	0.457	0.453		0.186	0.018	0.249	0.623	0.000
		X_P_	0.857	0.267	0.590	0.320		0.080	0.190	0.386	0.005
		bio17	-0.275	-0.309	0.034	-0.026	-0.069		0.129	0.074	0.003
		bio7	-0.711	-0.313	-0.398	-0.364	-0.162	0.128		0.347	0.043
Y_HγSS_					Total	→bio8	→bio15	→X_N_			
		bio8	0.863	1.020	-0.157		0.071	-0.228		0.720	0.000
		bio15	0.465	0.270	0.195	0.267		-0.072		0.178	0.005
		X_N_	-0.504	0.310	-0.814	-0.751	-0.063			-0.409	0.019
Y_DHγSS_											
		X_K_	0.451	0.451						0.203	0.021
Y_AC_											
		bio18	0.410	0.410						0.168	0.038
Y_AM_											
		bio3	0.703	0.703						0.494	0.011
Y_AA_											
		X_CEC_	0.651	0.651						0.424	0.013

Note: Y_γSS_, Y_AN_, Y_DHγSS_, …, Y_AA_ were performed in Table 1. Bio1, bio2, …, bio18 were performed in Fig. 3. X_CEC_-cationic exchange capacity (cmol(+)/kg), X_N_-total nitrogen content (g/kg), X_K_- total k content (g/kg), X_P_-total phosphorus content (g/kg), X_SOC_-soil organic carbon content (g/kg), X_BD_-soil bulk density (g/cm^3^), X_TH_-soil thickness (cm)

#### SEM of the effects of key environmental factors on amides contents

The structural equation model (SEM) is a statistical method for analyzing the relationship between variables based on the correlation coefficient or covariance matrix. The relationship between total amides contents, main amides components and environmental factors was expounded by SEM, and the SEM model fitting analysis with CMIN/DF < 1, RMSEA < 0.08 and AGFI > 0.9 was selected, which suggested that prediction results of the model were excellent.

**Fig. 4:**
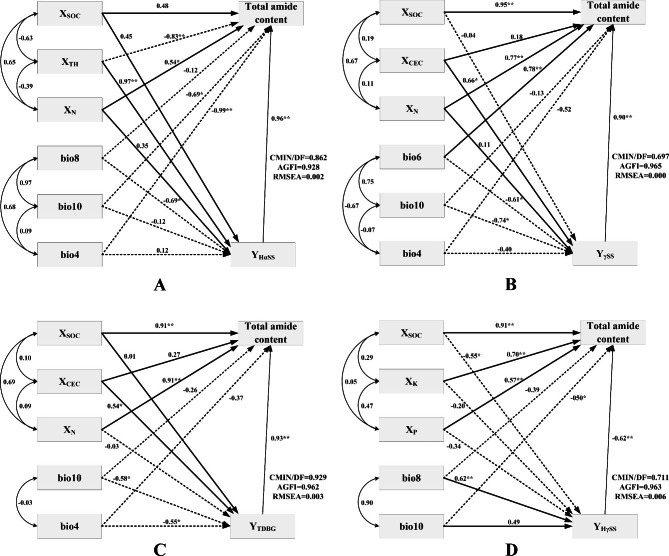
The structural equation model was used to test the relationship between environmental factors, amides and total amides content. **A** SEM of the effects of environmental factors and Y_HαSS_ on total amides content. **B** SEM of the effects of environmental factors and Y_γSS_ on total amides content. **C** SEM of the effects of environmental factors and Y_TDBG_ on total amides content. **D** SEM of the effects of environmental factors and Y_HγSS_ on total amides content. ** represents significant correlation at *p* < 0.01 level, * represents significant correlation at *p* < 0.05 level. Lines and dashed lines represented the linear relationship between the implicit and explicit variables of SEM, and the number was the path coefficient Note: Y_γSS_, Y_AN_, Y_DHγSS_, …, Y_AA_ were performed in Table 1. Bio1, bio2, …, bio18 were performed in Fig. 3. X_CEC_, X_N_, X_P_, X_K_, X_SOC_ were performed in Table 2.

Y_HαSS_, Y_γSS_, Y_TDBG_ and Y_HγSS_ had significant direct effects on total amide content, and the path coefficients were 0.96 (*P* < 0.01), 0.90 (*P* < 0.01), 0.93 (*P* < 0.01) and − 0.62 (*P* < 0.01), respectively (Fig. 4). X_SOC_ had a direct effect on total amide content, and the standardized path coefficient was 0.48. It also indirectly affected total amide content through Y_HαSS_, with an indirect path coefficient of 0.43 (0.45 × 0.96). X_TH_, bio10 and bio4 had significant negative effects on total amide content, and the path coefficients were − 0.83, -0.69 and 0.99, respectively; and the indirect path coefficients through Y_HαSS_ were 0.93 (0.97 × 0.96), -0.16 (-0.12 × 0.96) and 0.16 (0.12 × 0.96), respectively (Fig. 4A).

X_N_ and bio6 had direct effects on total amide content, and the standardized path coefficients were 0.77 (*P* < 0.05) and 0.78 (*P* < 0.01), respectively, and their indirect effects through Y_γSS_ on total amide content were 0.09 (0.11 × 0.90) and − 0.55 (-0.61 × 0.90), respectively. The indirect effect of bio10 through Y_γSS_ on total amide content was − 0.67 (-0.74 × 0.90) (Fig. 4B). The influence path coefficients of X_CEC_, bio10 and bio4 on total amide content through Y_TDBG_ were 0.50 (0.54 × 0.93), -0.54 (-0.58 × 0.93) and − 0.51 (-0.55 × 0.93), respectively (Fig. 4C). The indirect path coefficients of X_SOC_ and bio8 on total content through Y_HγSS_ were 0.34 (-0.55×-0.62) and − 0.38 (0.62×-0.62). X_N_ and X_P_ exhibited significant direct effects on total amides content, with the direct path coefficients of 0.70 (*P* < 0.01) and 0.57 (*P* < 0.01), respectively (Fig. 4D). It was found that the organic carbon content and total nitrogen in soil, the mean temperature of wettest quarter, mean temperature of warmest quarter, and temperature seasonality had significant effects on the single amide compound and total amide contents.

## Discussion

Different environments from different regions led to regional difference in plant effective components [24–26]. In our study, the amides contents in Chinese prickly ash peels from different habitats were 57.38 to 137.11 mg/g, with the average content of 88.92 mg/g. 13 amide compounds were identified, and the main amides were hydroxy-α-sanshool, hydroxy-β-sanshool and hydroxy-γ-sanshool, which accounted for 74.06% of the total amides, of which the average contents were 44.14, 10.77 and 7.18 mg/g, respectively. The amides contents in Chinese prickly ash peels from the high-altitude areas were significantly higher than that in the low altitude areas, with obvious altitude change trend. 26 Chinese prickly ash peels were clustered into high altitude-cool type and low altitude-warm type. Especially, the peels from Hanyuan County (132.586 mg/g), Maoxian County (117.562 mg/g), Wudu District (123.230 mg/g), Qin’an County (113.571 mg/g), Fengxian County (123.163 mg/g), had the highest amides contents. In addition, amides compounds contents in five samples (S22, S23, S24, S25, S26) that from the same region Shandong Province were differed from each other. Therefore, it suggested that the regions differences of amides content in Chinese prickly ash peels might be caused by climate and soil factors.

In addition, based on the PCA, HCA and OPLS-DA analysis of 13 amides compounds, Hydroxy-α-sanshool, γ-sanshool, ZP-amide N, hydroxy-γ-sanshool, bungeanool, dihydrosanshool and tetrahydrosanshool were the key differential amide components to distinguish peels from different locations. Based on the amides compounds contents, the Chinese prickly ash peels from different habitats were gathered into high altitude-cool type and low altitude-warm type. The contents of hydroxy-α-sanshool, γ-sanshool, ZP-amide N, hydroxy-γ-sanshool, dihydrosanshool, tetrahydrosanshool and bungeanool in Chinese prickly ash peels from high altitude areas (Hanyuan, Wudu, Qin’an and Fengxian) were higher. To meet the surging demand, the above regions could be selected for large scale cultivation of Chinese prickly ash, which was consistent with the actual producing area of Chinese prickly ash recognized as a good variety in China [2].

Climate factors could play an important role in regulating the synthesis and accumulation of the effective components in plants [27–29]. The correlation between 36 climatic factors and the flavonoids content of in the leaves of three Chinese prickly ash varieties showed that flavonoids displayed significant negative correlation with temperature and water vapor pressure, and positively correlated with wind speed. Climate factors had significant effects on the accumulation of afzelin, quercetin-3-O-glucoside, epicatechin and catechin, and had little effect on quercitrin, rutin and hyperoside [30]. Zheng et al. (2021) found that the volatile compounds contents were closely related to climate factors, and the effects of wind speed and annual mean temperature on volatile substances were greater than annual mean precipitation and annual sunshine duration [30].

In our research, γ-sanshool, ZP-amide N, dihydrosanshool, tetrahydrosanshool, bungeanool, hydroxy-α-sanshool and hydroxy-γ-sanshool were negatively correlated with annual temperature, max temperature of warmest month, mean temperature of wettest quarter and mean temperature of warmest quarter. The main amides in Chinese prickly ash peels (tetrahydrosanshool, bungeanool, hydroxy-β-sanshool, hydroxy-α-sanshool, hydroxy-γ-sanshool) were negatively correlated with the precipitation of wettest month and precipitation of warmest quarter. The study area belongs to the hydrothermal synchronization, indicating that high temperature was not conducive to amides formation in the development stage and mature stage. High temperature is the main meteorological disaster from coloring period to mature period. Excessive high temperature would reduce the activity of enzymes, affect the conversion of substances, and be not conducive to the conversion of sugar to downstream products, which was not conducive to the amides formation and accumulation. The amides were easy to be degraded in high humidity environment with air humidity over 80%. In short, low average temperature and less precipitation from coloring period to mature period were conducive to amides accumulation.

Soil was the basis for plant nutrient conservation and transformation, and plant growth and development and the effective component contents in plants were determined by soil fertility [31, 32]. The inorganic elements and organic carbon content in soil had a direct effect on the plants quality by affecting the nutrient absorption and the formation and accumulation of secondary metabolites [33, 34]. The main soil factors affecting the amides accumulation were soil organic carbon, available nitrogen, available phosphorus and available potassium. Most amides compounds were significantly positively correlated with the above soil factors and negatively correlated with soil bulk density. Soil organic carbon could provide sufficient nutrients (nitrogen, phosphorus, potassium) for the plant growth, increase the effectiveness of nutrients [35], reduce soil bulk density [36], increase pore volume and permeability, and improve soil storage, supply and water retention capacity [37]. In addition, organic carbon also has a good cation exchange capacity, the roots near the H^+^ or other cations exchange out at any time, supply pepper Na^+^, K^+^, Ca^2+^, Mg^2+^ and some trace nutrients [38, 39]. Potassium fertilizer could increase accelerate the transport of nutrients, photosynthesis intensity, promote the formation of starch and sugar, enhance the resistance and disease resistance of crops, and improve the absorption and utilization of nitrogen by crops [40, 41]. Nitrogen fertilizer is the main element of protein composition, and protein is also the basic material of cell protoplast composition [42]. Nitrogen fertilizer could promote the formation of protein and chlorophyll, increase chlorophyll content, increase leaf areas, thereby increasing crop yield and improving crop quality [43, 44]. Phosphorus is a component of cell wall, which can increase the aroma and numbness of Chinese prickly ash peels and enhance the color of fruit. Most amides compounds were significantly positively correlated with soil organic carbon, available nitrogen, available phosphorus and available potassium and negatively correlated with soil bulk density. Therefore, the amides yeild in Chinese prickly ash peels could be improved by scientific and effective fertilization to adjust the soil organic carbon and inorganic elements content of in soil.

Alkylamides are considered a combination of unsaturated fatty acids, valine, or phenylalanine. Alkylamide compounds have been proven to be the key components in Chinese prickly ash peels, but their biosynthesis pathways remain largely unknown. To fully elucidate the mechanisms by differetnt ecological factors induce amides synthesis and accumulation, the expression profiles of Chinese prickly ash peels cultured with different topographical conditions require investigation. Based on homologue searching, transcriptome analysis and ectopic expression, we would identified the candidate genes that may have contributed to amides synthesis in Chinese prickly ash peels under different ecological factors in the future experiments. Future experiments could cmobine with the present high-quality reference genome sequence leverage these findings to increase peels quality in Chinese prickly ash. Overall, this study provides valuable new insights into the amides accumulation underlying the responses of Chinese prickly ash peels to ecological factors.

## Conclusion

The total amides compounds contents in wild Chinese prickly ash peels from different regions were significantly different, with obvious altitude change trend. The contents of hydroxy-α-sanshool, γ-sanshool, ZP-amide N, hydroxy-γ-sanshool, dihydrosanshool, tetrahydrosanshool and bungeanool in peels from high altitude area were significantly higher than those in low altitude areas, and the numb-taste intensity was higher. The annual mean temperature, max temperature of warmest month, mean temperature of wettest quarter, mean temperature of warmest quarter, soil organic carbon, available nitrogen, phosphorus, and potassium were the main environmental factors affecting amides compounds accumulation. Different effects of climate and soil factors on amides contents were applied to optimized factor, select suitable agroclimatic zones for cultivation of selective varieties with high content of amide compounds. This aided in sites specific exploration of high amides yielding samples, and promoted the agriculture practices of Chinese prickly ash in regions with similar phytogeographical location.

## Electronic supplementary material

Below is the link to the electronic supplementary material.

Supplementary Material 1

## Data Availability

The datasets generated during and/or analyzed during the current study are available from the corresponding author on reasonable request.
