# Supplementary material for: Multi-environment evaluations across ecological regions reveal climate and soil effects on amides contents in Chinese prickly ash peels (Zanthoxylum bungeanum Maxim.)
Source: BMC Plant Biol. 2023 Jun 13;23:313. doi: 10.1186/s12870-023-04328-2 (PMC10262526; doi:10.1186/s12870-023-04328-2)
Supplement: Supplementary file 1 — Supplementary Material 1 [file 12870_2023_4328_MOESM1_ESM.docx]

**Supplement materials**

**Title**

Multi-environment evaluations across ecological regions reveal climate and soil effects on amides contents in Chinese prickly ash peels (*Zanthoxylum bungeanum* Maxim.)

**Author**

Tao Zheng^1,2,3^, Hai-tao Zeng^1,2,3*^, Bing-yin Sun^4^, Shu-ming Liu^5*^

^1^ Shaanxi University of Technology, College of Biology Science and Engineering, Hanzhong 723001, China

^2^ State Key Laboratory (Cultivation) of Qinba Mountains Biological Resources and Ecological Environment, Co-sponsored by the Ministry of Education and Shaanxi Province, Hanzhong 723001, China

^3^ Collaborative Innovation Center for Comprehensive Development of biological Resources in Qinba Mountains of Southern Shaanxi, Hanzhong 723001, China

^4^ Yangling Vocational &Technical College, Yangling 712100, China

^5^ Northwest Agriculture and Forestry University, College of Science, Yangling 712100, China

*Corresponding author

+E-mail: [zenghaitao@snut.edu.cn](mailto:zenghaitao@snut.edu.cn) (Hai-tao Zeng); [zhengtyhy@163.com](mailto:zhengtyhy@163.com) (Shu-ming Liu)

**Table S1** Chinese prickly ash peels geographic information of sampling localities.

| Provenance | Species | Procince and city | County | Longtitude | Latitude | Elevation(m) | Regions |
| --- | --- | --- | --- | --- | --- | --- | --- |
| S1 | Zanthoxylum bungeanum Maxim | Qinghai | Guide | 101.389 | 36.053 | 2188 | Northwest China |
| S2 | Zanthoxylum bungeanum Maxim | Sichuan | Hanyuan | 102.47 | 29.684 | 1791 | Southwest China |
| S3 | Zanthoxylum bungeanum Maxim | Sichuan | Hanyuan | 102.479 | 29.693 | 1869 | Southwest China |
| S4 | Zanthoxylum bungeanum Maxim | Qinghai | Xunhua | 102.528 | 35.833 | 1897 | Northwest China |
| S5 | Zanthoxylum bungeanum Maxim | Gansu | Linxia | 103.135 | 35.757 | 1789 | Northwest China |
| S6 | Zanthoxylum bungeanum Maxim | Sichuan | Maoxian | 103.787 | 31.782 | 1740 | Southwest China |
| S7 | Zanthoxylum bungeanum Maxim | Gansu | Wenxian | 104.665 | 33.048 | 1856 | Northwest China |
| S8 | Zanthoxylum bungeanum Maxim | Gansu | Wudu | 105.075 | 33.505 | 1420 | Northwest China |
| S9 | Zanthoxylum bungeanum Maxim | Gansu | Qin'an | 105.565 | 34.888 | 1621 | Northwest China |
| S10 | Zanthoxylum bungeanum Maxim | Gansu | Qin'an | 105.566 | 34.889 | 1646 | Northwest China |
| S11 | Zanthoxylum bungeanum Maxim | Shaanxi | Fengxian | 106.657 | 33.985 | 1011 | Northwest China |
| S12 | Zanthoxylum bungeanum Maxim | Shaanxi | Fuping | 109.211 | 34.983 | 787 | Northwest China |
| S13 | Zanthoxylum bungeanum Maxim | Shaanxi | Hengshan | 109.733 | 37.755 | 1195 | Northwest China |
| S14 | Zanthoxylum bungeanum Maxim | Shaanxi | Hancheng | 110.243 | 35.413 | 866 | Northwest China |
| S15 | Zanthoxylum bungeanum Maxim | Shaanxi | Hancheng | 110.246 | 35.409 | 880 | Northwest China |
| S16 | Zanthoxylum bungeanum Maxim | Shanxi | Yongji | 110.394 | 34.939 | 378 | North China |
| S17 | Zanthoxylum bungeanum Maxim | Henan | Lingbao | 110.541 | 34.384 | 540 | Central China |
| S18 | Zanthoxylum bungeanum Maxim | Shanxi | Ruicheng | 110.563 | 34.692 | 571 | North China |
| S19 | Zanthoxylum bungeanum Maxim | Henan | Lingbao | 110.571 | 34.519 | 545 | Central China |
| S20 | Zanthoxylum bungeanum Maxim | Shanxi | Jiaocheng | 112.12 | 37.564 | 847 | North China |
| S21 | Zanthoxylum bungeanum Maxim | Hebei | Shexian | 113.842 | 36.578 | 617 | North China |
| S22 | Zanthoxylum bungeanum Maxim | Shandong | Shanting | 117.586 | 35.05 | 201 | East China |
| S23 | Zanthoxylum bungeanum Maxim | Shandong | Shanting | 117.586 | 35.05 | 201 | East China |
| S24 | Zanthoxylum bungeanum Maxim | Shandong | Shanting | 117.586 | 35.05 | 201 | East China |
| S25 | Zanthoxylum bungeanum Maxim | Shandong | Laiwu | 117.589 | 36.502 | 330 | East China |
| S26 | Zanthoxylum bungeanum Maxim | Shandong | Laiwu | 117.589 | 36.502 | 330 | East China |

**Table S2** Data on the climate factors

| ID | bio1 | bio2 | bio3 | bio4 | bio5 | bio6 | bio7 | bio8 | bio9 | bio10 | bio11 | bio12 | bio13 | bio14 | bio15 | bio16 | bio17 | bio18 | bio19 | XSD | XMW |
| --- | --- | --- | --- | --- | --- | --- | --- | --- | --- | --- | --- | --- | --- | --- | --- | --- | --- | --- | --- | --- | --- |
| S1 | 6.779 | 14.325 | 36.543 | 884.126 | 24.400 | -14.800 | 39.200 | 17.000 | -4.733 | 17.000 | -4.733 | 407.000 | 91.000 | 1.000 | 97.638 | 239.000 | 4.000 | 239.000 | 4.000 | 2531.400 | 1.84 |
| S2 | 10.663 | 9.392 | 35.440 | 626.230 | 22.800 | -3.700 | 26.500 | 17.950 | 2.417 | 17.950 | 2.417 | 935.000 | 178.000 | 9.000 | 86.546 | 509.000 | 31.000 | 509.000 | 31.000 | 1123.500 | 2.24 |
| S3 | 8.929 | 9.408 | 35.370 | 626.844 | 21.100 | -5.500 | 26.600 | 16.283 | 0.733 | 16.283 | 0.733 | 898.000 | 172.000 | 10.000 | 85.041 | 475.000 | 33.000 | 475.000 | 33.000 | 1123.500 | 2.24 |
| S4 | 7.933 | 12.600 | 31.818 | 953.037 | 26.300 | -13.300 | 39.600 | 17.833 | -4.567 | 19.017 | -4.567 | 413.000 | 86.000 | 0.000 | 93.487 | 236.000 | 3.000 | 229.000 | 3.000 | 2470.900 | 3.06 |
| S5 | 8.196 | 11.208 | 29.809 | 933.667 | 25.800 | -11.800 | 37.600 | 17.950 | -4.017 | 19.050 | -4.017 | 420.000 | 90.000 | 0.000 | 90.306 | 237.000 | 4.000 | 223.000 | 4.000 | 2520.000 | 1.6 |
| S6 | 10.929 | 10.475 | 35.269 | 691.663 | 25.000 | -4.700 | 29.700 | 18.283 | 1.850 | 19.067 | 1.850 | 788.000 | 155.000 | 3.000 | 84.402 | 413.000 | 17.000 | 406.000 | 17.000 | 1549.400 | 3.8 |
| S7 | 8.667 | 9.800 | 32.558 | 730.875 | 22.800 | -7.300 | 30.100 | 16.517 | -0.833 | 17.367 | -0.833 | 693.000 | 129.000 | 3.000 | 80.196 | 349.000 | 16.000 | 337.000 | 16.000 | 1725.800 | 1.86 |
| S8 | 10.654 | 9.875 | 31.752 | 767.287 | 25.600 | -5.500 | 31.100 | 18.817 | 0.733 | 19.833 | 0.733 | 613.000 | 114.000 | 2.000 | 81.646 | 315.000 | 11.000 | 301.000 | 11.000 | 1623.600 | 1.63 |
| S9 | 8.242 | 9.933 | 29.216 | 869.477 | 24.900 | -9.100 | 34.000 | 17.433 | -2.933 | 18.683 | -2.933 | 591.000 | 109.000 | 4.000 | 80.345 | 309.000 | 16.000 | 288.000 | 16.000 | 1714.400 | 1.23 |
| S10 | 8.242 | 9.933 | 29.216 | 869.477 | 24.900 | -9.100 | 34.000 | 17.433 | -2.933 | 18.683 | -2.933 | 591.000 | 109.000 | 4.000 | 80.345 | 309.000 | 16.000 | 288.000 | 16.000 | 1714.400 | 1.23 |
| S11 | 11.713 | 9.242 | 29.154 | 832.445 | 27.300 | -4.400 | 31.700 | 20.650 | 1.117 | 21.767 | 1.117 | 705.000 | 130.000 | 4.000 | 78.699 | 370.000 | 16.000 | 320.000 | 16.000 | 2109.350 | 1.64 |
| S12 | 11.808 | 10.700 | 29.640 | 943.793 | 29.200 | -6.900 | 36.100 | 21.717 | -0.167 | 23.300 | -0.167 | 607.000 | 112.000 | 5.000 | 76.366 | 320.000 | 20.000 | 281.000 | 20.000 | 1247.400 | 1.86 |
| S13 | 8.679 | 12.908 | 29.880 | 1102.750 | 28.900 | -14.300 | 43.200 | 19.933 | -5.683 | 21.650 | -5.683 | 434.000 | 108.000 | 2.000 | 99.871 | 273.000 | 8.000 | 256.000 | 8.000 | 3235.700 | 2.16 |
| S14 | 11.333 | 11.117 | 29.964 | 964.757 | 28.900 | -8.200 | 37.100 | 21.433 | -1.050 | 22.883 | -1.050 | 545.000 | 109.000 | 4.000 | 82.147 | 302.000 | 14.000 | 267.000 | 14.000 | 2384.700 | 1.47 |
| S15 | 11.333 | 11.117 | 29.964 | 964.757 | 28.900 | -8.200 | 37.100 | 21.433 | -1.050 | 22.883 | -1.050 | 545.000 | 109.000 | 4.000 | 82.147 | 302.000 | 14.000 | 267.000 | 14.000 | 2384.700 | 1.47 |
| S16 | 13.775 | 11.767 | 30.884 | 991.621 | 31.600 | -6.500 | 38.100 | 24.100 | 1.083 | 25.667 | 1.083 | 558.000 | 108.000 | 5.000 | 74.953 | 288.000 | 18.000 | 255.000 | 18.000 | 2349.500 | 2.38 |
| S17 | 13.280 | 11.087 | 30.117 | 965.055 | 30.816 | -5.996 | 36.812 | 23.443 | 0.927 | 24.901 | 0.927 | 604.000 | 121.000 | 6.000 | 74.552 | 308.000 | 21.000 | 278.000 | 21.000 | 1932.600 | 2.46 |
| S18 | 13.196 | 11.442 | 30.675 | 964.821 | 30.800 | -6.500 | 37.300 | 23.350 | 0.867 | 24.833 | 0.867 | 585.000 | 117.000 | 6.000 | 75.342 | 302.000 | 20.000 | 270.000 | 20.000 | 2350.000 | 2.6 |
| S19 | 13.342 | 11.167 | 30.262 | 968.325 | 30.900 | -6.000 | 36.900 | 23.533 | 0.950 | 25.000 | 0.950 | 602.000 | 120.000 | 6.000 | 74.220 | 307.000 | 22.000 | 276.000 | 22.000 | 1932.600 | 2.46 |
| S20 | 9.950 | 13.350 | 32.246 | 1045.360 | 29.100 | -12.300 | 41.400 | 20.483 | -3.633 | 22.150 | -3.633 | 448.000 | 114.000 | 3.000 | 102.905 | 284.000 | 14.000 | 276.000 | 14.000 | 2344.300 | 1.69 |
| S21 | 11.333 | 11.417 | 29.654 | 1034.660 | 29.600 | -8.900 | 38.500 | 21.717 | -2.117 | 23.600 | -2.117 | 613.000 | 177.000 | 3.000 | 106.592 | 394.000 | 15.000 | 373.000 | 15.000 | 2129.000 | 1.26 |
| S22 | 13.075 | 10.233 | 28.585 | 992.807 | 29.700 | -6.100 | 35.800 | 24.800 | 0.233 | 24.800 | 0.233 | 716.000 | 205.000 | 9.000 | 102.604 | 457.000 | 36.000 | 457.000 | 36.000 | 1711.500 | 1.77 |
| S23 | 13.075 | 10.233 | 28.585 | 992.807 | 29.700 | -6.100 | 35.800 | 24.800 | 0.233 | 24.800 | 0.233 | 716.000 | 205.000 | 9.000 | 102.604 | 457.000 | 36.000 | 457.000 | 36.000 | 1711.500 | 1.77 |
| S24 | 13.075 | 10.233 | 28.585 | 992.807 | 29.700 | -6.100 | 35.800 | 24.800 | 0.233 | 24.800 | 0.233 | 716.000 | 205.000 | 9.000 | 102.604 | 457.000 | 36.000 | 457.000 | 36.000 | 1711.500 | 1.77 |
| S25 | 11.917 | 10.333 | 27.629 | 1041.760 | 29.200 | -8.200 | 37.400 | 24.100 | -1.650 | 24.100 | -1.650 | 715.000 | 226.000 | 6.000 | 112.622 | 468.000 | 23.000 | 468.000 | 23.000 | 2151.300 | 1.67 |
| S26 | 11.917 | 10.333 | 27.629 | 1041.760 | 29.200 | -8.200 | 37.400 | 24.100 | -1.650 | 24.100 | -1.650 | 715.000 | 226.000 | 6.000 | 112.622 | 468.000 | 23.000 | 468.000 | 23.000 | 2151.300 | 1.67 |

Note: bio1-Annual Mean Temperature (°C), bio2-Mean Diurnal Range (°C), bio3-Isothermality, bio4-Temperature Seasonality (°C), bio5-Max Temperature of Warmest Month (°C), bio6-Min Temperature of Coldest Month (°C), bio7-Temperature Annual Range (°C), bio8-Mean Temperature of Wettest Quarter (°C), bio9-Mean Temperature of Driest Quarter (°C), bio10-Mean Temperature of Warmest Quarter (°C), bio11-Mean Temperature of Coldest Quarter (°C), bio12-Annual Precipitation (mm), bio13-Precipitation of Wettest Month (mm), bio14-Precipitation of Driest Month (mm), bio15-Precipitation Seasonality (mm), bio16-Precipitation of Wettest Quarter (mm), bio17-Precipitation of Driest Quarter (mm), bio18-Precipitation of Warmest Quarter (mm), bio19-Precipitation of Coldest Quarter (mm), X_SD_-Annual sunshine duration(h), X_MW_-Mean wind speed (m/s).

**Table S3** Data on the soil factors

| ID | X_CEC_ | X_N_ | X_K_ | X_P_ | X_PH_ | X_SOC_ | X_BD_ | X_CF_ | X_TH_ |
| --- | --- | --- | --- | --- | --- | --- | --- | --- | --- |
| S1 | 18.07 | 0.79 | 17.95 | 0.57 | 8.42 | 8.06 | 1.34 | 9 | 130 |
| S2 | 19.73 | 2.14 | 20.71 | 0.69 | 6.02 | 21.08 | 1.173 | 10 | 97 |
| S3 | 22.57 | 2.33 | 20.38 | 0.67 | 6.06 | 26.19 | 1.029 | 20 | 87 |
| S4 | 18.17 | 2 | 17.81 | 0.59 | 8.39 | 16.55 | 1.241 | 9 | 172 |
| S5 | 18.18 | 1.92 | 17.65 | 0.63 | 7.84 | 18.43 | 1.16 | 12 | 114 |
| S6 | 18.08 | 1.75 | 20.6 | 0.72 | 7.05 | 16.93 | 1.246 | 20 | 101 |
| S7 | 17.84 | 1.95 | 20.42 | 0.66 | 7.56 | 24.25 | 1.15 | 8 | 104 |
| S8 | 17.43 | 2.09 | 20.52 | 0.69 | 7.97 | 20.37 | 1.248 | 11 | 133 |
| S9 | 16.92 | 2.18 | 21.89 | 0.69 | 8 | 19.41 | 1.276 | 6 | 198 |
| S10 | 13.45 | 2.17 | 20.2 | 0.72 | 8.12 | 18.23 | 1.273 | 4 | 203 |
| S11 | 16.73 | 1.53 | 20.66 | 0.71 | 7.93 | 22.72 | 1.316 | 7 | 126 |
| S12 | 14.27 | 0.82 | 17.84 | 0.58 | 7.89 | 8.02 | 1.345 | 14 | 160 |
| S13 | 11.75 | 0.45 | 14.8 | 0.46 | 8.08 | 5.89 | 1.337 | 6 | 214 |
| S14 | 14.72 | 0.92 | 17.93 | 0.62 | 8.15 | 9.01 | 1.311 | 4 | 197 |
| S15 | 15.12 | 0.94 | 18.09 | 0.58 | 8.19 | 9.32 | 1.318 | 4 | 200 |
| S16 | 14.1 | 0.72 | 19.4 | 0.68 | 8.72 | 8.35 | 1.456 | 1 | 195 |
| S17 | 15.47 | 0.72 | 15.67 | 0.54 | 6.65 | 7.84 | 1.134 | 19 | 191 |
| S18 | 16.26 | 0.82 | 15.25 | 0.57 | 8.04 | 8.63 | 1.386 | 3 | 218 |
| S19 | 12.93 | 0.78 | 17.26 | 0.66 | 8.46 | 7.8 | 1.36 | 3 | 203 |
| S20 | 14.31 | 0.77 | 16.44 | 0.48 | 8.32 | 6.88 | 1.328 | 18 | 152 |
| S21 | 16.7 | 1.09 | 16.92 | 0.53 | 7.76 | 11.13 | 1.305 | 38 | 68 |
| S22 | 15.51 | 1.00 | 16.18 | 0.46 | 7.03 | 8.44 | 1.336 | 17 | 98 |
| S23 | 15.51 | 1.00 | 16.18 | 0.46 | 7.03 | 8.44 | 1.336 | 17 | 98 |
| S24 | 15.51 | 1.00 | 16.18 | 0.46 | 7.03 | 8.44 | 1.336 | 17 | 98 |
| S25 | 14.64 | 0.98 | 16.57 | 0.41 | 6.98 | 8.94 | 1.322 | 21 | 96 |
| S26 | 14.64 | 0.98 | 16.57 | 0.41 | 6.98 | 8.94 | 1.322 | 21 | 96 |

Note: X_CEC_-cationic exchange capacity (cmol(+)/kg), X_N_-total nitrogen content (g/kg), X_K_- total k content (g/kg), X_P_-total phosphorus content (g/kg), X_SOC_-soil organic carbon content (g/kg), X_BD_-soil bulk density (g/cm^3^), X_TH_-soil thickness (cm).


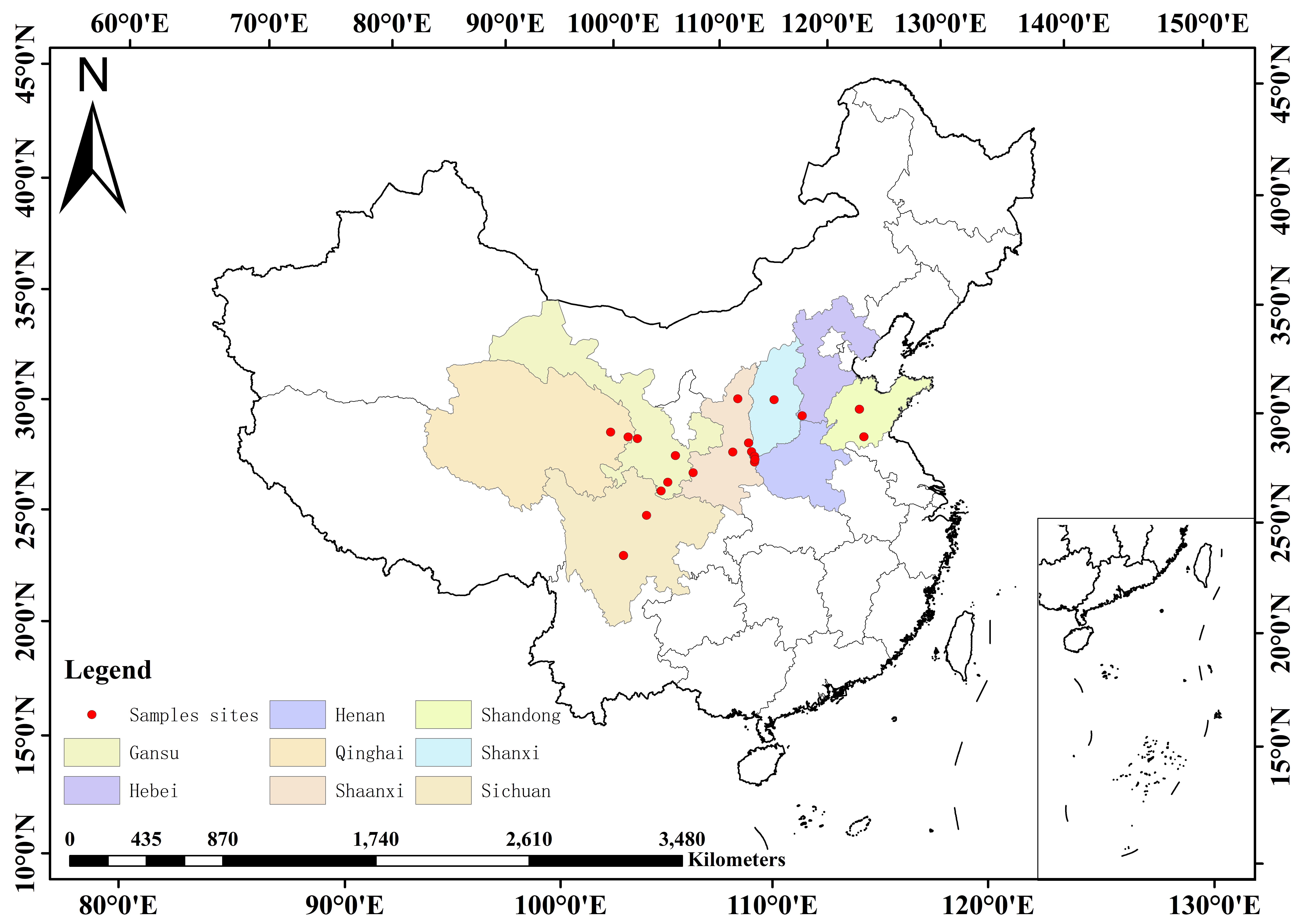


**Fig. S1** Sampling sites and geographical coordinate of Chinese prickly ash.


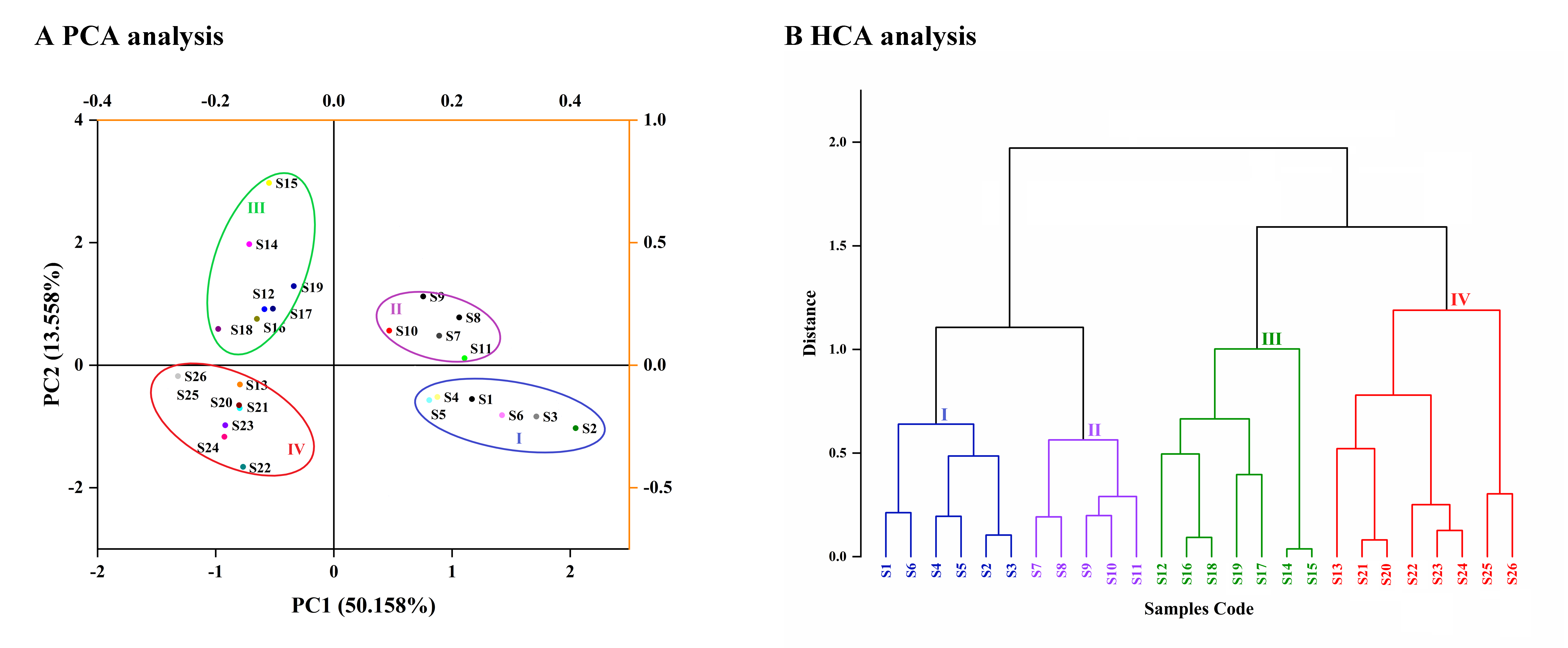


**Fig. S2** **A** PCA and **B** HCA analysis plot of Chinese prickly ash peels from different regions based on amides compounds contents.


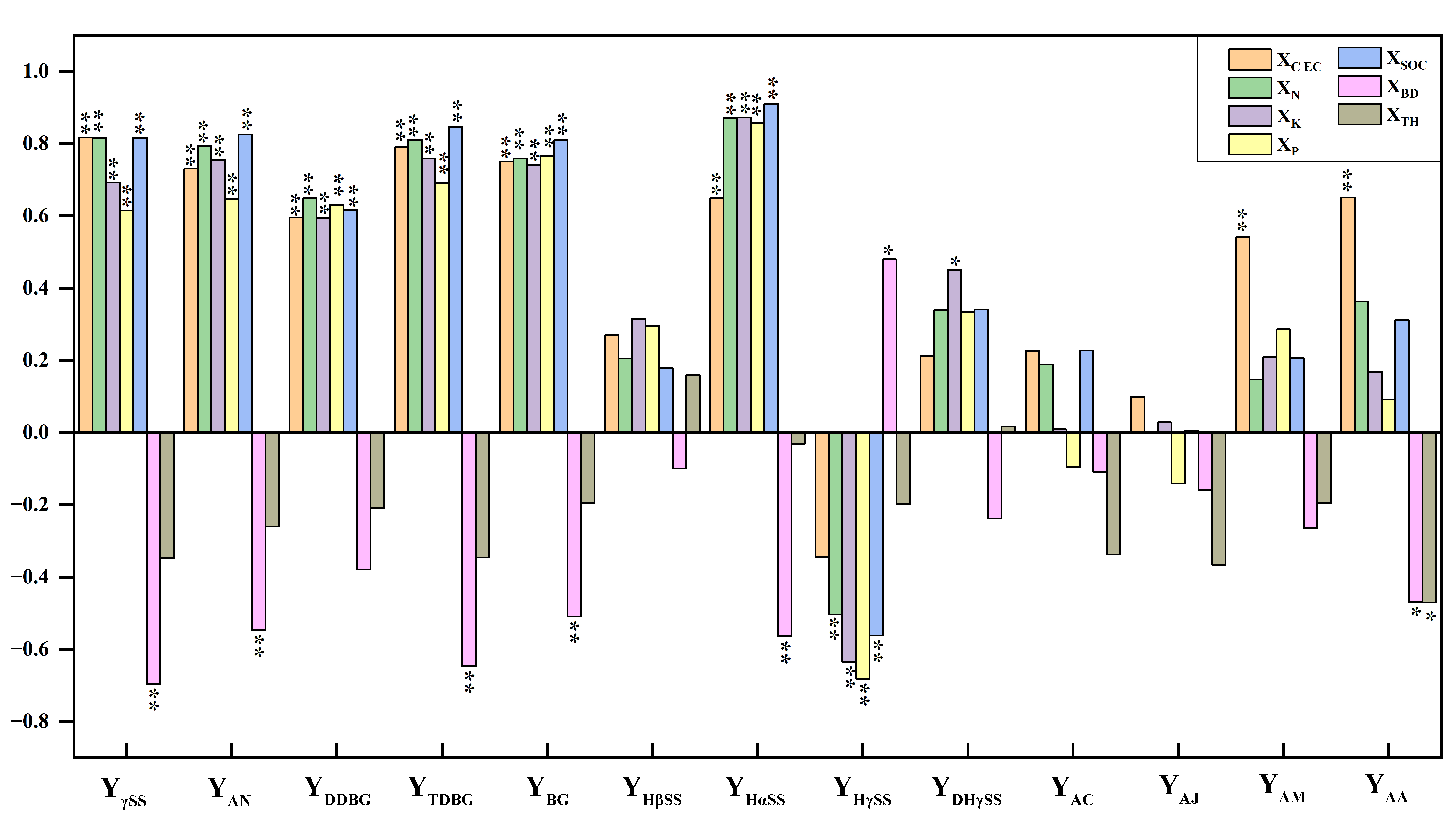


**Fig. S3** Correlation analysis results of soil factors and amides compounds.

Note: Y_γSS_, Y_AN_, Y_DHγSS_, …, Y_AA_ were performed in **Table 1**. X_CEC_-cationic exchange capacity (cmol(+)/kg), X_N_-total nitrogen content (g/kg), X_K_- total k content (g/kg), X_P_-total phosphorus content (g/kg), X_SOC_-soil organic carbon content (g/kg), X_BD_-soil bulk density (g/cm^3^), X_TH_-soil thickness (cm). ** represented significant difference at 0.01 level, and * represented significant difference at 0.05 level.
